# *Wolbachia* detection in *Aedes aegypti* using MALDI-TOF MS coupled to artificial intelligence

**DOI:** 10.1038/s41598-021-00888-1

**Published:** 2021-11-01

**Authors:** Antsa Rakotonirina, Cédric Caruzzo, Valentine Ballan, Malia Kainiu, Marie Marin, Julien Colot, Vincent Richard, Myrielle Dupont-Rouzeyrol, Nazha Selmaoui-Folcher, Nicolas Pocquet

**Affiliations:** 1 Institut Pasteur de Nouvelle-Calédonie, URE-Entomologie Médicale, 98845 Nouméa, New Caledonia; 2 Institut Des Sciences Exactes Et Appliquées, Université de Nouvelle-Calédonie, 98851 Nouméa, New Caledonia; 3 Institut Pasteur de Nouvelle-Calédonie, Groupe de Recherche en Bactériologie Expérimentale, 98845 Nouméa, New Caledonia; 4 Institut Pasteur, Direction Internationale, 75015 Paris, France; 5 Institut Pasteur de Nouvelle-Calédonie, URE-Dengue Et Autres Arboviroses, 98845 Nouméa, New Caledonia

**Keywords:** Biological techniques, Bioinformatics, Genetic techniques, Mass spectrometry

## Abstract

The mosquito *Aedes aegypti* is the major vector of arboviruses like dengue, Zika and chikungunya viruses. Attempts to reduce arboviruses emergence focusing on *Ae. aegypti* control has proven challenging due to the increase of insecticide resistances. An emerging strategy which consists of releasing *Ae. aegypti* artificially infected with *Wolbachia* in natural mosquito populations is currently being developed. The monitoring of *Wolbachia*-positive *Ae. aegypti* in the field is performed in order to ensure the program effectiveness. Here, the reliability of the Matrix‑Assisted Laser Desorption Ionization‑Time Of Flight (MALDI‑TOF) coupled with the machine learning methods like Convolutional Neural Network (CNN) to detect *Wolbachia* in field *Ae. aegypti* was assessed for the first time. For this purpose, laboratory reared and field *Ae. aegypti* were analyzed. The results showed that the CNN recognized *Ae. aegypti* spectral patterns associated with *Wolbachia*-infection. The MALDI-TOF coupled with the CNN (sensitivity = 93%, specificity = 99%, accuracy = 97%) was more efficient than the loop-mediated isothermal amplification (LAMP), and as efficient as qPCR for *Wolbachia* detection. It therefore represents an interesting method to evaluate the prevalence of *Wolbachia* in field *Ae. aegypti* mosquitoes.

## Introduction

Dengue, Zika and chikungunya diseases are globally emerging as a major public health problem in the world. These diseases attracted interest in recent years due to their increasing incidence and their expanding geographical range. Dengue fever is endemic in several countries with 390 million infections estimated per year^1^. Zika and chikungunya outbreaks also affected several regions and were responsible for high rates of morbidity and mortality^2–4^.

These diseases are caused by arboviruses that are transmitted to humans by vector mosquitoes, which one of the most widespread is *Aedes aegypti*. To date, no specific antiviral treatments are available. In addition, no vaccines for Zika and chikungunya are commercially available and the recently licensed dengue vaccine has limitations for its uses ^5,6^. Efforts to reduce the dengue, Zika and chikungunya incidence therefore rely on *Ae. aegypti* control. As mosquito control strategies are weakened due to the increase of insecticide resistances, the implementation of alternative and efficient strategies for arboviruses control are required^7^.

Recently, biocontrol strategy using *Wolbachia*, an endosymbiotic bacterium, was developed by various research groups^8–10^. This strategy is based on the ability of *Wolbachia* to reduce viral replication in *Ae. aegypti* and consists of releasing mosquitoes artificially infected with *Wolbachia* in natural *Ae. aegypti* populations. The World Mosquito Program (WMP; https://www.worldmosquitoprogram.org/) has implemented this strategy in several countries^11–14^, including New Caledonia (NC)^15^, which had released mosquitoes carrying *Wolbachia* across the main city of Noumea since 2019. Efficiency of this strategy relies on several features of the bacterium including: (1) maternal-inheritance infections resulting in *Wolbachia* maintain in progeny, (2) cytoplasmic incompatibility mechanism which contributes to uninfected mosquito population reduction and (3) significant inhibition of arboviruses transmission^16^.

However, for effective arboviruses control, *Wolbachia* infections should remain at high frequencies in *Ae. aegypti* populations^17^. Thus, the monitoring of infected *Ae. aegypti* through diagnostic testing remains an ongoing requirement to ensure the proper implementation and the effectiveness of the program.

In NC, two methods are currently used to diagnose *Wolbachia* infection in *Ae. aegypti*: the TaqMan™ quantitative Polymerase Chain Reaction (qPCR) and the LAMP assay. The qPCR remains the reference method^18^, this technic has the advantage of being sensitive and specific. Furthermore, it allows simultaneously: (1) *Ae. aegypti* species verification, (2) *Wolbachia* infection detection and (3) *Wolbachia* density quantification^19^. However, no information concerning the mosquito species is obtain in situations where *Ae. aegypti* internal control is not amplified (i.e., specific primers). In addition, the method is expensive and requires technical expertise.

The LAMP technique was described as suitable for high throughput application due to its good sensitivity and specificity^18,20^. Compared to qPCR, the result interpretation is more accessible, and the technique allowed a rapid screening of samples. Nevertheless, the main disadvantage of LAMP method is the use of a single target nucleic acid test, allowing only the detection of *Wolbachia*. In some situations, especially when samples are degraded by the trapping method, a confusion with another *Aedes* species can occur at the identification step, this can lead to a false-negative interpretation and an underestimation of the *Wolbachia* frequency.

Matrix-Assisted Laser Desorption Ionization-Time of Flight Mass Spectrometry (MALDI-TOF MS) has recently emerged for identification tool in medical entomology using mass spectra databases. This technique allows simultaneous arthropod species identification and pathogens detection in a single experiment^21–23^. In 2015, Yssouf et al*.* showed the ability of MALDI-TOF MS to identify the tick species and to detect the presence of *Rickettsia* pathogen, an intracytoplasmic bacterium, in a single assay^24^. In addition, in terms of required expenses, MALDI-TOF MS is much cheaper than the qPCR and LAMP techniques.

Current advances in machine learning approaches could complement and enhance performance of the MALDI-TOF MS to analyze mosquito spectra. For example, it was used to recognize MALDI-TOF MS spectral patterns associated with *Anopheles* ages, blood-feeding and *Plasmodium* infection^25^. Machine learning methods is a domain includes all supervise classification methods. Particularly, we use deep learning method like convolutional neural network (CNN) for field-mosquitoes recognition which is a method based on neural network. CNN^26,27^ is an architecture of artificial neural network based on multilayers perceptron. These are usually fully connected neural networks. Each neuron of a layer is connected to all neurons of the next layer.

To our knowledge, no studies have investigated CNN application on *Wolbachia* detection in mosquitoes. Therefore, the aim of this work was to investigate the use of MALDI-TOF MS and the CNN for *Wolbachia* detection, in the context of implementing an arbovirus control strategy based on the use of this endosymbiotic bacterium. Our ultimate goal was to develop this tool for a rapid and reliable monitoring of *Wolbachia* infection rate in *Ae. aegypti* collected from the field.

## Methods

### Biological material

During this study, laboratory-reared *Ae. aegypti* were used, including mosquitoes infected with *Wolbachia* (*w*Mel-strain) and uninfected specimens (Wild Type-strain, or WT). They were raised in insectarium until adult stage, with a temperature of 28 °C ± 1 °C and relative humidity of 80% ± 10%. Adult mosquitoes were subsequently harvested.

In addition, males and females *Ae. aegypti* collected from the field were also used during this study. Field sampling was conducted in Noumea, NC, from December 2019 to April 2020 (Supplementary Table S1) using BG-sentinel® traps (Biogents, Regensburg, Germany). This sampling was assessed during the first phase of releases in Noumea (i.e., September 2019 to June 2020). A maximum trapping duration of 24 h was set to avoid mosquito proteins degradation as previously described^28^.

All mosquitoes were killed by freezing at − 20 °C and were morphologically identified to the species level using morphological keys^29–32^. Then, *Ae. aegypti* mosquitoes (males and non-blood fed females) were dissected into two body parts: (1) head and thorax (without legs and wings), and (2) abdomen, then preserved at − 80 °C between 6 and 72 days before MALDI-TOF analyses. Preservation at − 80 °C prevents protein degradation and guarantees good mass spectra acquisition even after long storage durations^28^.

### qPCR and LAMP analysis

DNA extracts was carried out, using abdomen, for both laboratory and field mosquitoes. Extraction was performed using DNA blood and tissues kit (Qiagen, Hilden, Germany) according to the manufacturer’s instruction.

DNA extracted was subsequently amplified by multiplex qPCR using specific primers targeting *Ae. aegypti* house-keeping *Rps17* and *Wolbachia* specific *wsp* genes, as previously described^18^. Briefly, qPCR conditions were: 0,25 µM each of forward and reverse primers, 0,1 µM each of probes, 1X Lightcycler 480 Probes Master reaction mix, 1 µL of target DNA in a total reaction volume of 10 µl. Thermal cycling was performed using Roche LightCycler 480 (Roche Life Science, USA), with 1 cycle at 95 °C for 5 min for pre-incubation, followed by 45 cycles of 95 °C for 10 s, 60 °C for 15 s and 72 °C for 1 s for amplification followed by a final cooling step of 40 °C for 10 s^18^. In addition, *Wolbachia* density in mosquitoes, which are an indication of the ratio of *Wolbachia* DNA relative to *Ae. aegypti* DNA in each mosquito, were calculated using relative quantification algorithm in the LightCycler 480 software^19^.

For each field-mosquito, extracted DNA were also subjected to LAMP assay. For this experiment, three pairs of primers were used as described by Gonçalves et al.^18^. Samples were amplified at 65 °C for 30 min and held at 12 °C until scoring^18^. LAMP technic provides a drop of pH when DNA amplification occurs, modifying the color of the mix: pink indicates negative, orange indicates equivocal and yellow indicates positive sample. Results were interpreted by direct observation and photos were taken with a tablet computer for data storage.

### Protein extraction protocol

For each mosquito, protein extraction was performed using head and thorax according to the protocol previously published^28^. Abdomen was excluded from analysis in order to increase the reproducibility and quality of spectra and reduce the bias caused by the midgut microbiota^33^. Briefly, these were put into individual microtubes and rinsed with 1 mL of 70% ethanol for 60 s, followed by 1 mL of distilled water for 60 s. The remaining water was then eliminated using a micropipette and left to evaporate. Three metal beads, 15 µL of acetonitrile (50%) and 15 µL of formic acid (70%) were subsequently added to the microtube. Each sample was subsequently homogenized (automated method) using a MagnaLyser, version 1.1 (Roche, Mannheim, Germany) with 3 cycles of 30 s at a frequency of 3000 rpm. After homogenization, 1 µL of sample was deposited directly on a steel MALDI plate (Bruker Daltonics, Wissembourg, France) and allowed to dry before adding another 1 µL of the same sample over the first spot^28^. To create the reference main spectrum pattern (MSP), a total of 8 spots were spotted of the same sample^28^. Conversely, each sample intended to be queried against these reference spectra were deposited in duplicate, as suggested by previous work^28^. Matrix solution was also loaded in duplicate onto each MALDI-TOF plate in order to control matrix quality.

### MALDI-TOF MS analysis

For MALDI-TOF MS analysis, 12 independent experiments were realized. For each experiment, the MALDI-TOF plate contained mosquitoes belonging to the two categories, *w*Mel and WT. The mass spectrometer Microflex LT/SH (Bruker, France), with sample throughput of 200 samples per hours, was used for analysis. Measurements were performed with flexControl software, version 3.3 (Bruker) with detection in the linear positive-ion mode at a laser frequency of 60 Hz and laser power between 40 and 50%. Each spectrum obtained, ranging from 2000 to 20,000 Da, corresponds to an accumulation of 240 laser shots from the same spot performed in 6 regions.

During this study, laboratory reared *Ae. aegypti* were used to create the MSPs. Specifically, a total of 10 MSPs were created, comprising 5 *w*Mel MSPs and 5 WT MSPs (Table 1). The created mass spectra library was evaluated for mosquito species identification and *Wolbachia* detection during a blind test, using Bruker Daltonics algorithm.

**Table 1:** Overview of mosquitoes used for MALDI-TOF MS analysis.

	MSPs creation	Blind testing [number of spectra obtained]
Laboratory strain *w*Mel	5	30 [60]
Laboratory strain WT	5	30 [60]
Field-mosquito *w*Mel	–	56 [102]
Field-mosquito WT	–	138 [249]
Total	10	254 [471]

For the blind test analysis, laboratory mosquitoes including 30 *w*Mel-strain and 30 WT-strain were analyzed. In addition, 194 mosquitoes from the field were included in analyses (Table 1). For this, the spectra acquired from the duplicate of each sample was compared to the created MSPs. Then, the MALDI Biotyper Compass software version 3.1 (Bruker Daltonics) calculates Log-score value (LSV) ranging from zero to three, reflecting the similarity between sample spectra and MSP. As previously stated, a LSV ≥ 1.8 support the mosquito species identification^28,34^.

To find biomarkers of *Wolbachia* infection, all laboratory and field-mosquito spectra were imported into the FlexAnalysis software (Bruker Daltonics). These were visually inspected in order to compare protein profile of the *w*Mel and WT mosquitoes.

### CNN training for mass spectra classification

**Figure 1:**
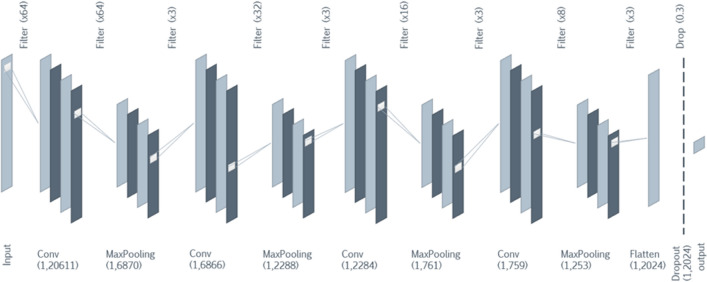
Architecture of the convolutional neural network used for *Wolbachia* detection in *Ae. aegypti*.

**Figure 2:**
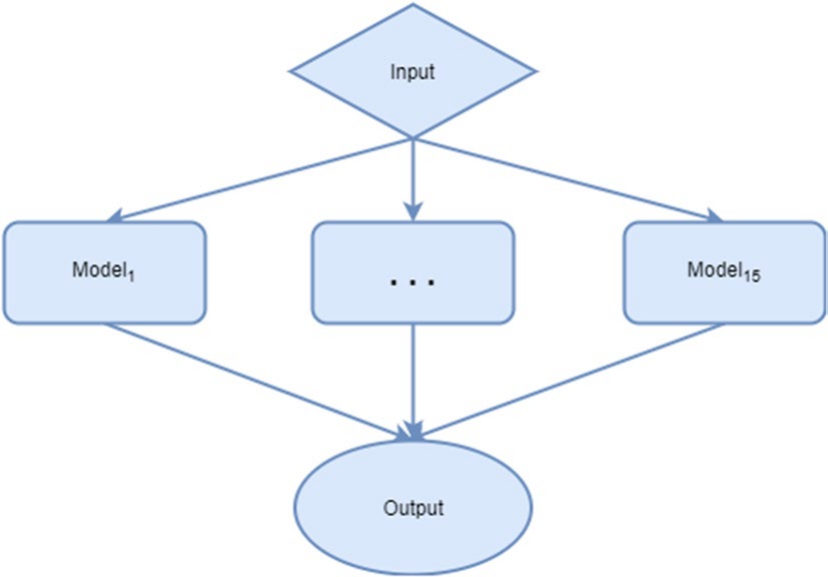
Architecture of the ensemble learning used during analysis.

All spectra of field and laboratory mosquitoes generated during this study were exported from the FlexAnalysis software (Bruker Daltonics). The spectra used are the pre-processed spectra (smoothing by baseline subtraction), then they have been normalized so that the values vary in the same ranges. In addition, the architecture only accepts signals of the same size (length). A dimension upscaling of all the spectra was performed by adding null values at the end of each signal for those that had a size smaller than the largest spectra. The architecture^26^ of the CNN model used is composed of 4 pairs of layers alternating Convolution and Maxpooling layers and ends with a Flattening layer connected to a Fully Connected layer, Dropout, and finally the Output layer (Fig. 1). An ensemble learning architecture was achieved to improve the performance of the classification (Fig. 2). The ensemble learning architecture is composed of 15 CNN as described previously, each and every model has been trained on different parts of the data with random initialization, which help generalize and make robust classification. We store the performance indicators of every model. Then, a voting system is set up based on a newly developed equation which gives the final classification. This process improves the overall performance of the classification.

Each spectrum acquired from the duplicate of one mosquito, was considered as a single input (considered as data augmentation). For 37 mosquitoes, only one spectrum was obtained from the duplicate. Thus, a total of 471 spectra were included in the analysis. This consisted of 120 spectra of laboratory-mosquitoes and 351 spectra of field-specimens. In order to avoid biases, multiple scenarios have been considered. Firstly, to avoid overfitting and to generalize the model, we use the k-fold cross-validation^35,36^ technique. This consists in randomly dividing the dataset into k subgroups (here, k = 5). One group (i.e., test dataset) was used for the model validation, while the k − 1 groups (i.e., training dataset) were dedicated to training the model. Then, K-fold results were then averaged to produce a single estimate of the performance indicators (accuracy, precision, recall, etc.). Furthermore, to overcome the unbalanced class problem, an upsampling of the minority classes has been carried out.

Finally, based on the output of the test dataset, we performed a quantitative evaluation of the ensemble learning performance to recognize *Ae. aegypti* spectral patterns associated with *Wolbachia* infection. Then, Sensitivity (Se), specificity (Sp), positive predictive value (PPV), negative predictive values (NPV) and classification accuracy were computed.

### Feature extraction

The CNN is a deep learning model based on an artificial neural network with several layers connected to each other. Each layer (which operates as a black box) receives and processes information from the previous layer. This information contains the features calculated at each layer that allowed the discrimination of classes. So, each layer improves its learning by refining the features previously calculated. To extract the features, we therefore calculate the frequency of a given peak carried out through the layers (using the feature maps given by the CNN of each layer). Thus, features were extracted in order to identify the spectra region used by CNN to discriminate infected and uninfected *Ae. aegypti*. The data containing the major mass points within the spectra used by the CNN to discriminate infected and uninfected *Ae. aegypti,* and their corresponding intensities were exported. This was analyzed with R software (R Core Team (2017). R: A language and environment for statistical computing. R Foundation for Statistical Computing, Vienna, Austria).

### Comparison of performances of LAMP, MALDI-TOF MS protein profiling and MALDI-TOF MS coupled to CNN

The performance of LAMP and MALDI-TOF MS protein profiling to detect *Wolbachia* in field-mosquitoes was compared to the reference qPCR analysis performance. For this, sensitivity (Se), Specificity (Sp), Positive Predictive Value (PPV), Negative Predictive Value (NPV) and classification accuracy were computed. We also performed a quantitative evaluation of the classification performance of CNN when using MALDI-TOF MS spectra. This was based on the rates of correctly classified spectra in the positive and negative labelled classes, according to the qPCR reference technique.

### Ethics approval and consent to participate

Mosquito collection authorization was granted by authorities from the South Province of New Caledonia (ordinance No. 1415–2019/ARR/DENV).

## Results

### Mosquito species confirmation and *Wolbachia* detection with the reference qPCR method

During this study, the morphological identification of field and laboratory-mosquitoes was confirmed using qPCR technic. The *Rps17* gene was correctly amplified for all samples, confirming that they belonged to the *Ae. aegypti* species.

In addition, *Wolbachia* screening was realized on the laboratory-strain using qPCR method. The *Ae. aegypti* infected with *Wolbachia* (*w*Mel-strain) showed amplification of the *wsp* gene. The mean *Wolbachia* density was of 15 (95% CI 12–19) copies of *w*Mel per copy of the *Rps17* gene. Conversely, no amplification of *wsp* gene was observed for the WT mosquitoes.

A total of 194 *Ae. aegypti* from the field were analyzed with the qPCR technic. All were positive for *Rps17* gene. Results showed 56 *Wolbachia*-infected mosquitoes. The mean of *Wolbachia* density was of 30 (95% CI 19–41) copies of *w*Mel per copy of the *Rps17* gene. Conversely, qPCR result showed that 138 field-*Ae. aegypti* were not infected by *Wolbachia*.

### *Wolbachia* detection with LAMP technic

All mosquitoes from the field were also analyzed using LAMP method. Among the 56 *Wolbachia* infected mosquitoes (results obtained with the reference qPCR), the bacterium was detected in 45 individuals*.* Equivocal and negative results were obtained for 4 and 145 mosquitoes, respectively.

### MALDI-TOF MS results

During the query of the reference mass spectra library, 98% (n = 60) and 90% (n = 194) of respectively laboratory-reared and field mosquitoes were correctly identified at species level (LSV ≥ 1.8). For *Wolbachia* detection, we observed mismatches. Indeed, 47% (95% CI 28–66%) and 70% (95% CI 56–81%) of respectively *w*Mel laboratory-reared and field-collected mosquitoes incorrectly matched with WT MSPs. Similarly, 27% (95% CI 12–46%) and 16% (95% CI 10–23%) of respectively WT laboratory-reared and field mosquitoes incorrectly matched with *w*Mel MSPs.

**Figure 3:**
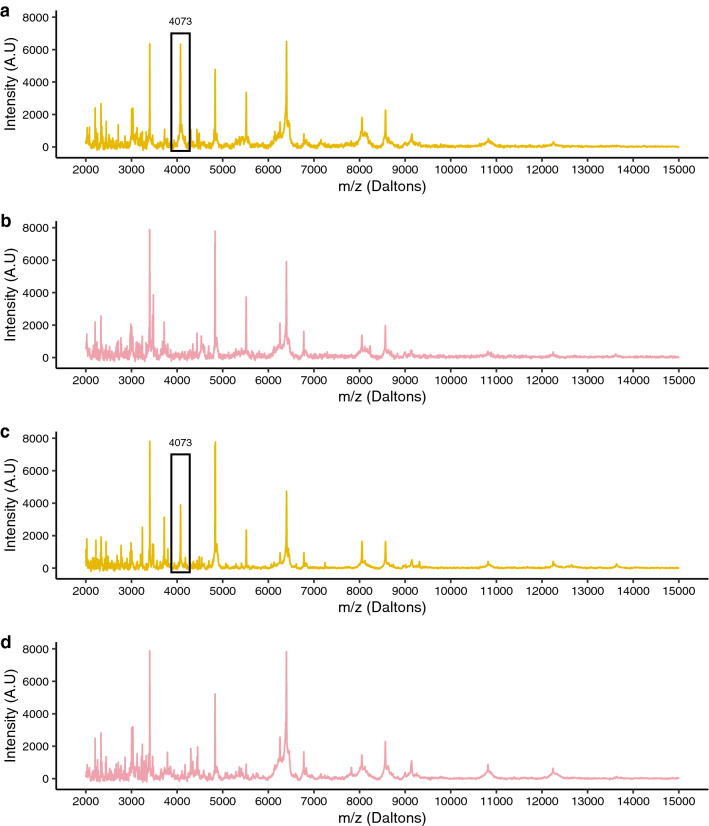
Comparison between the spectra of *Ae. aegypti* infected with *Wolbachia* and *Ae. aegypti* uninfected using FlexAnalysis software. (**a**): spectra of infected *Ae. aegypti* from the laboratory. (**b**): spectra of uninfected *Ae. aegypti* from the laboratory. (**c**): spectra of infected *Ae. aegypti* from the field. (**d**): spectra of uninfected *Ae. aegypti* from the field. Abbreviations: A.U, arbitrary unity; m/z, mass-to-charge ratio.

Using protein profiling, a peak at 4073 ± 3 Da and high intensity (i.e., > 2000 a.u) was detected in 100% of *w*Mel laboratory *Ae. aegypti* spectra. Conversely, this peak was considered as absent in all WT laboratory-mosquito spectra (Fig. 3). When observing the spectra of field-specimens, the peak at 4073 ± 3 Da was also detected in 43 mosquito spectra among the 56 *Wolbachia* infected field-mosquitoes and was present in the spectrum of one WT-mosquito (qPCR results). This peak was considered as absent in the other 150 field-specimen spectra.

### Distinction between spectra of *Wolbachia* infected and uninfected *Ae. aegypti* with CNN

With the architecture of the proposed CNN model, we first used a dataset of 471 spectra in order to train and validate the model (one or two spectra per specimen, depending on the number of spectra obtained for the two deposits per specimen on the MALDI-TOF plate). For the laboratory-strain, the results showed 55 *Wolbachia-*infected spectra and 65 uninfected spectra. A discordance between the results within the duplicate was observed for 3 mosquitoes. Regarding the result of field-mosquitoes, 100 infected spectra and 251 uninfected spectra were found. For 6 mosquitoes, a discordance between the results within the duplicate was also observed. Moreover, when using the CNN, the bacterium was detected in 9 of 11 mosquitoes with low *Wolbachia* densities.

The performance of the CNN to discriminate spectra of *Wolbachia*-infected and uninfected mosquitoes was also evaluated using laboratory-reared and field specimens. We report the confusion matrices provided by the CNN separately for laboratory-strain and field mosquitoes (Table 2).

**Table 2:** Confusion matrices of field and laboratory mosquitoes’ classification with CNN on MALDI-TOF MS spectra.

Laboratory mosquitoes	Predicted positive	Predicted negative	Field mosquitoes	Predicted positive	Predicted negative
Actual Positive	27	3	Actual Positive	52	4
Actual Negative	0	30	Actual Negative	2	136

### Analysis of *Ae. aegypti* spectra profiles according to CNN results

**Figure 4:**
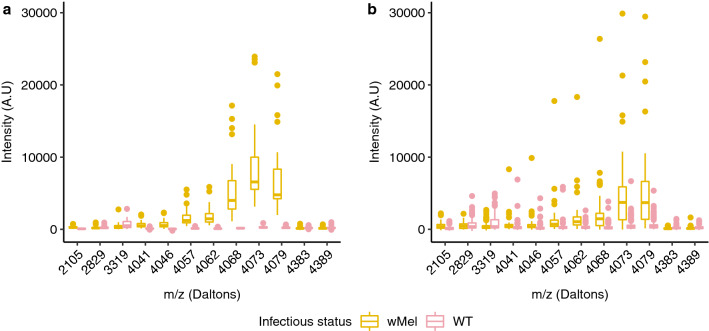
Boxplot showing the 12 mass points of distinct m/z (Daltons) considered over 85% by the CNN to discriminate *Ae. aegypti* infected and uninfected with *Wolbachia*. (**a**): results obtained for laboratory-reared mosquitoes (*w*Mel: n = 30; WT: n = 30). (**b**): results obtained for field mosquitoes (*w*Mel: n = 56; WT: n = 138). The boxplot colors correspond to the infection status of the mosquitoes determined by qPCR (yellow: *w*Mel; pink: WT). Abbreviations: A.U, arbitrary unity; m/z, mass-to-charge ratio.

For spectra profiling, using the features extracted from the CNN, arbitrary thresholds are defined to help with discrimination. This allows us to make a comparison between the calculation made manually and what the CNN allows us to do automatically. We can see that the CNN finds other possibilities to discriminate between the spectra representing infected or uninfected mosquitoes when the important peaks do not have the same intensity range. This confirms the robustness of the CNN model. Feature extraction allowed to observe a total of 60 mass points (i.e., biomarkers) within the spectra which were over 80% used by the CNN to discriminate the spectra of *Wolbachia* infected and uninfected *Ae. aegypti* (Supplementary Fig. S1). The observation of the result showed that the single peak visually observed when using the FlexAnalysis software (Bruker Daltonics) was a set of mass points forming a bell curve around 4073 ± 3 Da. Regarding the spectra areas used at more than 85% by the CNN, a total of 12 mass points within the spectra were used, including 7 points located in the 4073 ± 3 Da region for the laboratory and field mosquito spectra (Fig. 4a,b, respectively).

### Comparison of performances of LAMP, MALDI-TOF MS protein profiling and MALDI-TOF MS coupled to CNN

**Figure 5:**
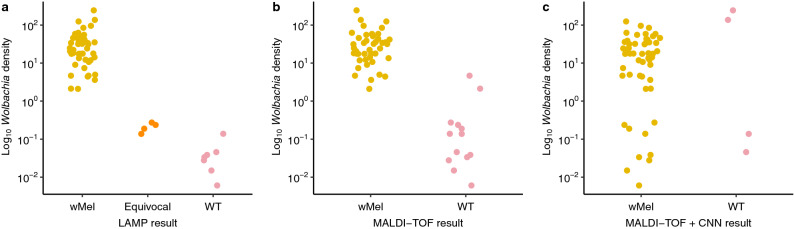
Classification of field *w*Mel *Ae. aegypti* according to their *Wolbachia* density by LAMP, MALDI-TOF MS protein profiling, and MALDI-TOF MS coupled to CNN. (**a**): results obtained with LAMP assay. (**b**): results obtained with MALDI-TOF MS protein profiling. (**c**): results obtained with MALDI-TOF MS coupled to CNN. Each dot represents one field *Ae. aegypti* detected as positive for *Wolbachia* by qPCR (n = 56). Y axis corresponds to the decimal logarithm of *Wolbachia* density according to the qPCR results. In the X-axis, *w*Mel corresponds to the true positive results, equivocal (only for LAMP) and WT correspond to the false negative results.

Relative to the qPCR reference method, no LAMP false positive was observed when analyzing field-mosquitoes. Conversely, false negatives results (i.e., equivocal and negatives) were observed for 11 mosquitoes. For these mosquitoes, low *w*Mel densities (i.e., < 1) were found (Fig. 5a). According to these results, a sensitivity of 80% (95% CI 69–90%), a specificity of 100% (95% CI 97–100%) and an accuracy of 94% (95% CI 90–97%) were achieved for LAMP method (Table 3).

**Table 3:** Comparison of LAMP, MALDI-TOF MS profiling, and MALDI-TOF MS coupled to CNN performances when analyzing field-mosquitoes (n = 194).

Technic	Se [95% CI]	Sp [95% CI]	PPV [95% CI]	NPV [95% CI]	Accuracy [95% CI]
LAMP	80% [68–90]	100% [97–100]	100%	93% [88–95]	94% [90–97]
MALDI-TOF	77% [64–87]	99% [96–100]	98% [86–100]	91% [87–94]	93% [88—96]
CNN on MALDI-TOF	93% [89–96]	99% [97–100]	96% [94–99]	97% [95–99]	97% [94–99]

Abbreviations: Se, sensitivity; Sp, specificity; PPV, positive predictive value; NPV, negative predictive value.

The performance indicators were calculated in comparison to the results of the gold standard qPCR technique.

Concerning the MALDI-TOF technic, when we consider the presence of the peak at 4073 ± 3 Da as an indicator of *Wolbachia* presence in *Ae. aegypti*, one false positive result was observed for field-mosquitoes, while 13 false negatives were found. With the exception of two specimens, low *w*Mel densities (i.e., < 1) were observed for these false negative specimens (Fig. 5b). With only a visual analysis of the spectra, a sensitivity of 77% (95% CI 64–87%), a specificity of 99% (95% CI 96–100%) and an accuracy of 93% (95% CI 88–96%) were obtained (Table 3).

For the MALDI-TOF MS coupled to CNN, based on the results obtained for one spectrum per mosquito, two false positive results were observed for field-mosquitoes. In contrast, the number of false negatives was greatly reduced compared to MALDI-TOF alone, with only four false negatives results. Indeed, most mosquitoes with a low density of *Wolbachia* (i.e., < 1) were better classified by the CNN (Fig. 5c). The result showed a sensitivity of 93% (95% CI 89–96%), a specificity of 99% (95% CI 97–100%) and an accuracy of 97% (95% CI 94–99%).

## Discussion

The release of *Ae. aegypti* mosquitoes artificially infected with the endosymbiotic bacterium *Wolbachia* in the field is one of the most promising ongoing intervention against arboviruses. The World Health Organization has recently published that this strategy demonstrates public health value against dengue virus^37^. However, in order to evaluate the effectiveness of this program, the prevalence of *Ae. aegypti* infected with *Wolbachia* must be assessed through screening trapped mosquitoes. In this study, we present the first assessment of the accuracy of the MALDI-TOF MS and artificial intelligence to detect the presence of *Wolbachia* in field *Ae. aegypti*.

In *Ae. aegypti* artificially infected with *Wolbachia,* the bacterium is localized throughout most tissues such as the fat body, midgut, muscle, nervous tissue and Malpighian tubules^38^. The bacterium is also present in the salivary glands, which are located at the anterior portion of the thorax, and is more abundant in their ovaries, located in the abdomen^9^. During this study, the abdomen was not used for MALDI-TOF MS analysis to reduce bias caused by potential traces of blood-meal and microbiota in the spectra^34^. This part of the body was used for qPCR and LAMP assays, while the head and thorax were tested for MALDI-TOF analysis. In parallel, the legs of these mosquitoes were also tested on MALDI-TOF but did not allow good classification of *Wolbachia*-infected and uninfected mosquitoes (Raw Data are provided in Supplementary Table S1).

The result of MALDI-TOF analysis showed that the routine analysis, using the MALDI-compass software (Bruker Daltonics), consisting in comparing the mosquito spectra with the reference spectra (i.e., MSPs), did not allow a successful distinction between *Wolbachia*-infected and uninfected *Ae. aegypti*. Indeed, mismatches between mosquito’ infectious status were found. Previous work consisting in the detection of filariae in *Ae. aegypti* has shown a variation of correct identification rates according to the compartment tested when querying of a reference database^22^. In contrast, other works have shown the 100% reliability of the MALDI-TOF database queries to detect the presence of bacterium such as *Rickettsia* in tick legs and haemolymph^24,39^. These contradictory results suggest that care should be taken when using only database query for microorganism detection in arthropods.

The observation of the spectral profile of laboratory reared *Ae. aegypti* allowed to visually detect a peak at 4073 Da in 100% of *w*Mel mosquitoes, which was absent in WT specimens. When considering this peak as specific biomarker of *Wolbachia*-infected mosquitoes, the performance of the MALDI-TOF MS to detect the bacterium in field-mosquitoes was evaluated. Results showed a sensitivity of 77% and most false-negative samples observed had low *Wolbachia* densities, with the exception of two field *Ae. aegypti*. These findings highlighted that despite the sensitivity of 77% of the MALDI-TOF MS when using only the FlexAnalysis software, this method allows the detection of *Wolbachia* in the field-mosquitoes when the density of the bacteria is high (> 1); these mosquitoes could have strong pathogen blocking and could show a complete cytoplasmic incompatibility and maternal transmission^7,16,40^.

When analysing the MALDI-TOF spectra with the CNN, an improvement of the performance was found. Indeed, the results showed a sensitivity of 93%, a specificity of 99%, and an accuracy of 97%. In addition, among the 11 mosquitoes with low *Wolbachia* density, 9 mosquitoes were considered as positive with the CNN. It should be noted that mismatches were observed in CNN results between the duplicate of six individuals from the field, regardless of their infectious status (4 WT and 2 *w*Mel-mosquitoes according to qPCR results). Despite this, results demonstrated that the CNN could recognize the spectral pattern of *Wolbachia*-infected and uninfected *Ae. aegypti*, allowing a robust classification according to their infectious status. Similarly, other authors have shown the ability of artificial intelligence to predict *Anopheles* aging, their blood feeding status and *Plasmodium* infection when using MALDI-TOF MS spectral profile^25^. These proofs of concept extend the field of applications of MALDI-TOF MS coupled with artificial intelligence in medical entomology. As performant as the reference qPCR, this technique represents an interesting complementary method for the monitoring of *Ae. aegypti* infected with *Wolbachia* in the field.

Furthermore, the results obtained during the feature extraction showed that the set of mass points forming a bell curve around 4073 Da stood out from the other mass points considered for the classification. These mass points could correspond to *Wolbachia*-specific proteins. These could also correspond to a response of the mosquitoes to the bacterial infection. Indeed, previous work has shown that some proteins such as defensin A, an antimicrobial peptide could be implicated in dengue virus inhibition when analyzing the haemolymph of transgenic strains of *Ae.* *aegypti*^41^. Interestingly, according to other authors, the estimated mass of defensin A was of 4075 Da^42^. Thus, this could correspond to the bell curve (i.e., around 4073 Da) considered by the CNN during our study. However, complementary experiments are needed to test this hypothesis.

The performance of MALDI-TOF protein profiling and coupled to CNN to detect *Wolbachia* in field-mosquitoes was compared to the reference qPCR method. However, as the LAMP technique is currently used to monitor the progress of the *w*Mel *Wolbachia* establishment in Noumea, NC, we also compared the MALDI-TOF and LAMP performance. Results showed a robustness of the LAMP technique, with a sensitivity of 80% which was similar than the sensitivity of MALDI-TOF protein profiling, but lower than the MALDI-TOF coupled with the CNN. When comparing our results with those from a previously reported assay^18^, our study showed a lower LAMP sensitivity value. However, in this previous paper, equivocal samples have been excluded from their analysis^18^. In our case, equivocal mosquitoes were considered as negative samples to allow the comparison of the three techniques. Moreover, we also found that the false-negative and equivocal samples observed during LAMP analysis corresponded to field-specimens with low *Wolbachia* density. Previous works have shown that the reduction of *Wolbachia* density in mosquito result in a weakness of cytoplasmic incompatibility and maternal transmission^7,40^ and potentially reduced arbovirus blockage^16^. Thus, the LAMP false-negatives and equivocal can be considered as uninfected mosquitoes due to the potential loss of traits necessary for the strategy success.

All these findings suggest that the MALDI-TOF coupled to CNN is reliable for *Wolbachia* detection in *Ae. aegypti* from the field. Unlike the LAMP technique, it allows *Ae. aegypti* species verification. This information on mosquito species is crucial to avoid a false-negative or false-positive interpretation in case of non-*Ae. aegypti* mosquitoes. Moreover, the cost of reagents and consumables required for MALDI-TOF MS is about ten times cheaper than for qPCR and LAMP (Supplementary Table S2). While the initial cost of the MALDI-TOF instrument is high, the cost savings on reagents can offset the expenditure within a few years. Finally, in our study, with experienced personal, MALDI-TOF MS analysis (starting from protein extraction to spectra acquisition) can be realized in about 2h30min for one plate (i.e., 96 samples), which is shorter than the time required for qPCR and LAMP analysis (more than 6 h and 5 h, respectively, starting from DNA extraction to DNA amplification).

Our overall results also underlined the need to include field-mosquitoes in analysis to ensure the application of this new tool through field studies. Indeed, we found a higher mean of *Wolbachia* density in field-mosquitoes compared to laboratory strain, but with a higher variability (Supplementary Table S1). Conversely, the variation of intensities of peaks considered by CNN of field-mosquitoes was less important compared to the profile of laboratory mosquitoes. This could be related to the higher variability of field mosquitoes or a slight degradation of proteins due to trapping^28^.

This study has potential bias and limitations. The possible limitation of this work was the unavailability of information on the identity and functions of the proteins corresponding to the mass points used by the CNN during analysis. However, the distinction between spectra of *Wolbachia-*infected and uninfected *Ae. aegypti* was completed despite the non-identification of these peaks. It will be interesting in the future to carry out analysis using high-end mass spectrometers such as LC–MS/MS systems or MALDI-TOF/TOF in order to have more information on the proteins corresponding to these mass points.

In addition, if the features used by the CNN to classification are consistent with a mosquito's response to *Wolbachia* infection, it is possible that this response changes slightly depending on the genetic background of the mosquito population tested. It would therefore be important to verify the effectiveness of this CNN in another country implementing this strategy.

The current CNN has good results for the classification between *Wolbachia*-infected and non-infected mosquitoes, but it does not return any information on *Wolbachia* density. As mentioned above, mosquitoes with a low *Wolbachia* density (i.e., < 1) lose partially their ability to block viral replication^16^. In the future, in order to better monitor the implementation of *Wolbachia*-based strategies with MALDI-TOF, it would be useful to develop a multi-class CNN that would allow the distinction between uninfected, low *Wolbachia* density and high *Wolbachia* density mosquitoes.

Finally, as previously shown, a trapping duration of more than 24 h could lead to protein degradation and alter the MALDI-TOF results^28^. The daily sampling of traps in the field represents a particular challenge for the use of this tool to detect *Wolbachia* in field *Ae. aegypti* adults. To circumvent this limitation, *Wolbachia* detection could be assessed using eggs or larvae mosquitoes collected by ovitraps or in their breeding site. In the future, the accuracy of MALDI-TOF MS coupled to CNN for *Wolbachia* detection in larvae mosquitoes should be therefore tested.

## Conclusions

In conclusion, the MALDI-TOF MS coupled to CNN is a reliable tool for the field-monitoring of *Ae. aegypti* infected with *Wolbachia*. It allows detection of the bacterium, with high sensitivity and specificity. The use of artificial intelligence in MALDI-TOF MS spectra analysis is an emerging approach in medical entomology. It will be interesting to apply this tool in other entomological fields such as the pathogen detection in arthropod vectors or determination of the age structure of field-mosquito population.

## Supplementary Information

Supplementary Information 1.

Supplementary Information 2.

Supplementary Information 3.

## Data Availability

Data are available from the corresponding author upon reasonable request.
