# Supplementary material for: Wolbachia detection in Aedes aegypti using MALDI-TOF MS coupled to artificial intelligence
Source: Sci Rep. 2021 Nov 1;11:21355. doi: 10.1038/s41598-021-00888-1 (PMC8560810; doi:10.1038/s41598-021-00888-1)
Supplement: Supplementary file 1 — Supplementary Information 1. [file 41598_2021_888_MOESM1_ESM.pdf]

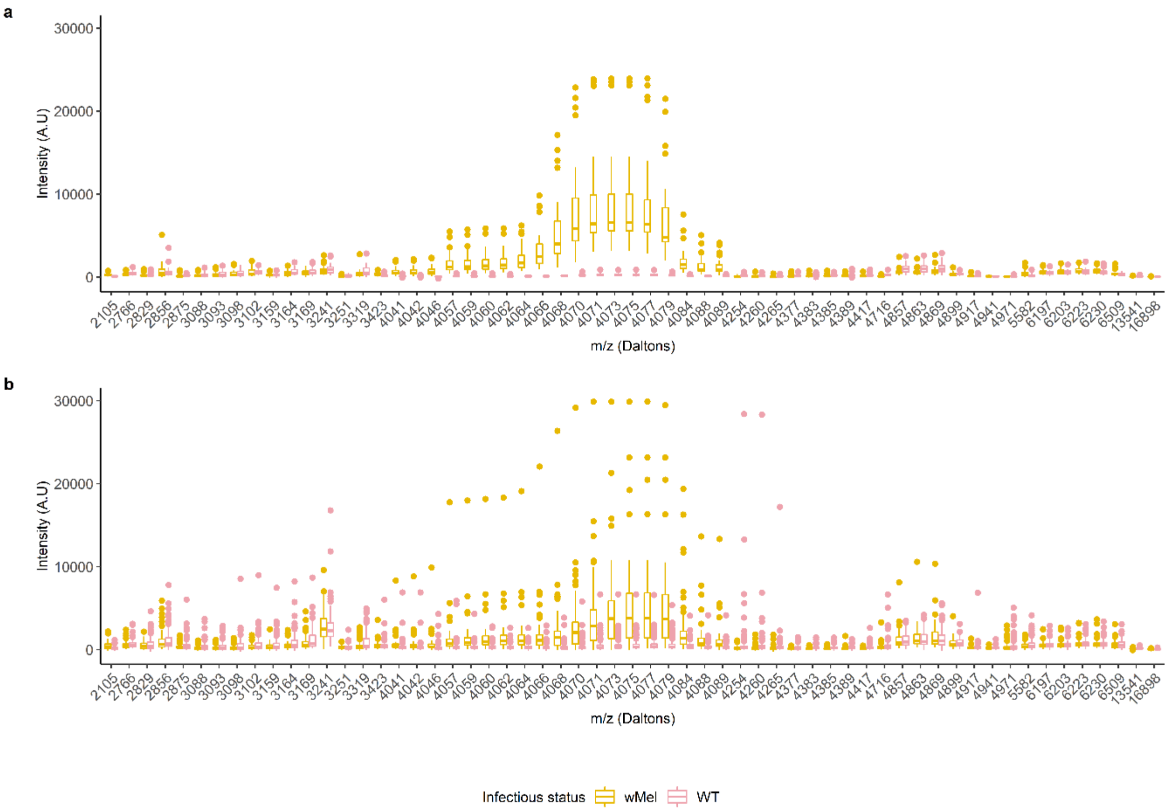

Supplementary Figure S1. Boxplot showing the 60 mass points of distinct m/z (Daltons) considered over 80% by the CNN to discriminate *Ae. aegypti* infected and uninfected with *Wolbachia*. a: results obtained for laboratory-reared mosquitoes (*w*Mel: n = 30; WT: n = 30). b: results obtained for field mosquitoes (*w*Mel: n = 56; WT: n = 138). The boxplot colors correspond to the infection status of the mosquitoes determined by qPCR (yellow: *w*Mel; pink: WT). Abbreviations: A.U, arbitrary unity; m/z, mass-to-charge ratio.
