# Supplementary material for: Wolbachia detection in Aedes aegypti using MALDI-TOF MS coupled to artificial intelligence
Source: Sci Rep. 2021 Nov 1;11:21355. doi: 10.1038/s41598-021-00888-1 (PMC8560810; doi:10.1038/s41598-021-00888-1)
Supplement: Supplementary file 3 — Supplementary Information 3. [file 41598_2021_888_MOESM3_ESM.pdf]

	MALDI-TOF	qPCR	LAMP
Machine' cost	170,000 €	5,000 €	3,000 €
Cost of reagent and consumable for 1 sample	0.61 €	1.43 €	2.29 €
Amortized machine' cost over 5 years (a)	34,000 €	1,000 €	600 €
Annual cost of reagent and consumable; for 60,000 mosquitoes (b)	36,807 €	85,714 €	137,143 €
Total annual cost (a + b)	70,807 €	86,714 €	137,743 €

Supplementary table S2. Detail on the annual cost of diagnosing field-mosquitoes using the 3 techniques. The estimated number of mosquitoes (*i.e.,* 60,000) is based on the approximate number of mosquitoes analyzed in New Caledonia per year. Costs are based on prices in New Caledonia.
